# Measuring Health-Related Quality of Life With Multimodal Data: Viewpoint

**DOI:** 10.2196/35951

**Published:** 2022-05-26

**Authors:** Ieuan Clay, Francesca Cormack, Szymon Fedor, Luca Foschini, Giovanni Gentile, Chris van Hoof, Priya Kumar, Florian Lipsmeier, Akane Sano, Benjamin Smarr, Benjamin Vandendriessche, Valeria De Luca

**Affiliations:** 1 Digital Medicine Society Boston, MA United States; 2 Cambridge Cognition Cambridge United Kingdom; 3 MIT Media Lab Cambridge, MA United States; 4 Evidation Health San Mateo, CA United States; 5 University of Padua Padua Italy; 6 imec Leuven Belgium; 7 Rune Labs San Fransisco, CA United States; 8 pRED Hoffmann-La Roche Basel Switzerland; 9 Department of Electrical and Computer Engineering Rice University Houston, TX United States; 10 Department of Bioengineering and Halicioglu Data Science Institute University of California, San Diego San Diego, CA United States; 11 Byteflies Antwerp Belgium; 12 Novartis Institutes for Biomedical Research Basel Switzerland

**Keywords:** digital measures, quality of life, machine learning, digital health, digital product, digital wellness, digital therapeutics, digital therapy, multimodal technology, drug development, care delivery, data integration

## Abstract

The ability to objectively measure aspects of performance and behavior is a fundamental pillar of digital health, enabling digital wellness products, decentralized trial concepts, evidence generation, digital therapeutics, and more. Emerging multimodal technologies capable of measuring several modalities simultaneously and efforts to integrate inputs across several sources are further expanding the limits of what digital measures can assess. Experts from the field of digital health were convened as part of a multi-stakeholder workshop to examine the progress of multimodal digital measures in two key areas: detection of disease and the measurement of meaningful aspects of health relevant to the quality of life. Here we present a meeting report, summarizing key discussion points, relevant literature, and finally a vision for the immediate future, including how multimodal measures can provide value to stakeholders across drug development and care delivery, as well as three key areas where headway will need to be made if we are to continue to build on the encouraging progress so far: collaboration and data sharing, removal of barriers to data integration, and alignment around robust modular evaluation of new measurement capabilities.

## Introduction

The field of digital health has become a multibillion dollar market, powering a paradigm shift by enabling the continuous capture of multimodal data including activity, sleep, vital signs, and contextual information. Novel machine learning applications are pioneering the conversion of these multimodal data into measures for health-related quality of life (QOL)–relevant symptoms like fatigue [1], stress [2], and depression [3,4]. These insights have the potential to enable improved care delivery [5] and a deeper understanding of patients’ lived experiences and better, more personalized medicines. However, important barriers remain to realize these benefits, both in technical and social aspects of real-world adoption.

On July 27, 2021, as part of the IEEE-EMBS International Conference on Biomedical and Health Informatics jointly organized with the 17th IEEE-EMBS International Conference on Wearable and Implantable Body Sensor Networks [6], a workshop was held on “Measuring Quality of Life with Multimodal Data.” The workshop was divided into two sessions, the first focusing on disease detection and the second focusing on the measurement of well-being. Abstracts from the keynotes and talks are presented in Table 1; this meeting report summarizes key discussion points, relevant literature, and finally a vision for the immediate future, including how multimodal measures can provide value to stakeholders across drug development and care delivery, as well as three key areas where headway will need to be made if we are to continue to build on the encouraging progress so far: collaboration and data sharing, removal of barriers to data integration, and alignment around robust modular evaluation of new measurement capabilities.

**Table 1:** Talk titles and abstracts for all presented work.

Speaker			Affiliation		Title		Abstract		Citations and further reading
**Session 1: disease detection**									
	Author LF	Evidation Health		PGHD^a^: a new ally for public health		PGHD from smartphones, wearables, and other sensors have the potential to transform the way health is measured, with broad-ranging applications from clinical research to public health and health care at large. This talk will survey examples of applications of PGHD across therapeutic areas, including post-op monitoring, screening for cognitive impairment, and a particular focus on public health applications for flu and COVID-19 detection and quantification. Finally, I will discuss lessons learned in translating PGHD research into benefits for the individual, with emphasis on the importance of evaluating analytics performance (eg, AUROC^b^, sensitivity, and specificity) within a specific context of use of a real-world application.		[7,8]	
	Author FL	Roche Pharma Research and Early Development, F. Hoffmann-La Roche Ltd		Digital health technology tools and quality of life: examples from current studies in neurological disorders		In recent years, DHTTs^c^ such as smartphones and wearables are becoming an integrated part of clinical research. Augmented by novel often AI^d^-powered signal processing, they enable continuous and precise measurements of disease symptoms. It is therefore becoming important to link these measures to the different aspects of QOL^e^ of patients to make them meaningful tools for drug decision-making. In this talk, I will highlight examples from DHTTs we are developing for neurological disorders such as Parkinson disease, multiple sclerosis, and Huntington disease. Leveraging active testing and patient questionnaires accompanied by passive continuous monitoring in daily life, these tools offer rich sets of data. General signal processing and dedicated machine learning/AI solutions are used to unlock these data sets and relate them back to standard clinical scores of disease severity. I will show how resulting measures relate to patients’ self-perceived health-related quality of life, how DHTTs used during COVID-19–induced lockdowns can offer new insights on QOL perception, and how we envision strengthening the link between novel sensor measurements and patient-relevant symptoms and impacts.		[9,10]	
	Author BV	Byteflies		Leveraging multimodal sensor data to assess complex chronic conditions at home		Byteflies’s Sensor Dot platform enables continuous acquisition of physiologic and behavioral data. We leverage this multimodal data to move diagnostic tests typically performed in a specialized environment to the home of the user and to make longitudinal assessments of chronic conditions possible. In both cases, an understanding of the continuous changes in activities of daily living is crucial for safe and accurate clinical interpretation of the data. In this talk, I will discuss EpiCare@Home, a remote epileptic seizure monitoring solution built on top of the Byteflies platform.		[11,12]	
	Author GG	Department of Neuroscience, University of Padua, Italy; SENSEDAT Srl, Padua, Italy		Unsupervised wearable and machine learning approach to identify depression, anxiety, and stress physiological phenotypes		Background: Anxiety and depression are defined with clinical interviews in RCTs^f^, possibly inflating intervention’s/placebo’s effects. We here introduce an algorithm to identify anxiety and depression with wearable-measured physiological biomarkers. Objectives: To validate a machine learning–based algorithm using wearable unsupervised measurements of the autonomic nervous system and physiological parameters to classify clinical anxiety and depression according to validated questionnaires. Methods: Included were physically healthy workers from the general population wearing an arm-band wearable device equipped with photoplethysmogram and electrodermal activity sensors for 24 hours. Participants answered validated self-report questionnaires for mental health, including PSS-10^g^, GAD-7^h^, and PHQ-9^i^. Wearable recordings were subject to artifact removal, signal preprocessing, and split in 30-second blocks for which physiological indexes and related features were extracted. A feature fusion approach was implemented together with the C5.0 machine learning algorithm, which was run on 70% randomly selected preprocessed blocks, and on the remaining 30% for external validation. Coprimary outcomes were anxiety (GAD-7≥10), and depression (PHQ-9≥10). Results: We included 95 participants (yielding 237,778 monitoring blocks), 47.7% females, mean age 37.2 (SD 15.5) years. Overall, 13.7% had anxiety, 12.6% had depression, and 7.4% had both. In the main sample, the wearable machine learning algorithm showed excellent accuracy for coprimary outcomes, namely, AUC^j^=0.928 for anxiety and AUC=0.959 for depression. Discussion: Limitations of the study include self-report questionnaires to assess primary outcomes and its cross-sectional nature. Potential implications of this work include biomarker-based inclusion criteria in RCT testing interventions for anxiety and depression, as well as screening and monitoring tools of mental health issues in the general population. Further studies should replicate the proposed algorithm against structured interview-based diagnoses with different wearable devices on clinical samples, possibly with a longitudinal design.		[13-15]	
**Session 2: measuring well-being**									
	Author AS	Rice University		Multimodal sensor data analysis and modeling for health and well-being		Digital phenotyping and machine learning technologies have shown a potential to measure objective behavioral and physiological markers, provide risk assessment for people who might have a high risk of poor health and well-being, and help make better decisions or behavioral changes to support health and well-being. I will introduce a series of studies, algorithms, and systems we have developed for measuring, predicting, and supporting personalized health and well-being. I will also discuss challenges, learned lessons, and potential future directions in health and well-being research.		[2,16]	
	Author BS	UCSD^k^ Department of Bioengineering and the Halicioglu Data Science Institute; Oura		The future of health and wellness discovery is democratic		Engineered solutions for personal data generation (wearable sensors, apps, etc) and analysis are proliferating rapidly, but health services served by these technologies continue to lag behind. Complexity in human diversity stymies algorithm generalizability and hampers successful wide adoption of any specific solution. We propose that efforts at expanding engagement in discovery will achieve two complementary goals: (1) promote mapping of biological diversity beyond demography and genetics into physiology and behavior so that algorithms can be developed on empirically determined subpopulations, and (2) fertilize natural experiments that will reveal communities sharing needs and goals, for whom solutions can then be tailored. Efforts to expand engagement may enable a virtuous cycle where iterative improvement and expansion in precision wellness technologies go from intractable to standard in personal, community, and clinical settings.		[17,18]	
	Author FC	Cambridge Cognition; Department of Psychiatry, University of Cambridge		Characterizing fatigue using digital technologies		Fatigue is both common and burdensome across a range of patient groups. The manifestation of fatigue is complex, comprising both subjective and objective changes to cognitive and physical performance, and is determined by a range of factors, including sleep, mood, time of day, competing demands, and environmental context, as well as disease-specific variables. These factors, and consequently the patient’s experience of fatigue, vary with time, meaning that infrequent in-clinic assessments are likely to be of limited sensitivity. Given this complexity, we have been interested in exploring the potential role of digital technologies in capturing and characterizing fatigue, particularly the impact of fatigue on cognitive performance, across a range of clinical conditions. This talk will focus on methods of data collection such as brief active assessments, voice capture, and passive data from wearable technology, and describe insights these data provide us into this complex symptom.		[3,19]	
	Author CvH	Connected Health Solutions, imec; OnePlanet Research Center		Nanoelectronics and AI for our (and our planet’s) health		We are faced with global challenges related to health, food, sustainability, and the environment. While these are formidable challenges, they also represent a substantial opportunity to improve people’s lives on a global scale while at the same time creating new economic opportunities. We are convinced nanoelectronics and digital technologies are the key tools for disruptive solutions. With that purpose in mind, the OnePlanet Research Center was created as a multidisciplinary collaboration between imec, Radboud University Medical Center, and Wageningen University & Research. In OnePlanet, we apply nanoelectronics and analytics innovations to solve problems related to personalized health, personalized nutrition, mental well-being, sustainable food production, and reduced environmental impact. The sensors and data innovations are working toward the creation of digital twins for prevention, early detection, or interception of disease.		[20,21]	
	Author SF	MIT Media Lab		Monitoring well-being using longitudinal passive data		The boundaries between the consumer and clinical device markets are becoming leaner every year. This trend is driven by a number of factors including consumer demand for ubiquitous and constantly accessible health care; increased presence of chronic conditions (eg, high blood pressure, diabetes, depression, and obesity); and a corresponding need for preventive health care, an increasingly aging global population, availability of cost-effective wearable technology, and remote access to storage and computation resources. This trend enables substantial opportunities for providing health care services to larger populations at lower cost. It will also pave the way to personalized medicine where prevention, diagnosis, and treatment of a disease can be tailored to individuals’ characteristics and behavior. In this presentation, recent developments of wearable technologies at MIT Media Lab and their application to the diagnosis of mental health diseases and overall well-being are discussed.		[22,23]	

^a^PGHD: person-generated health data.

^b^AUROC: area under the receiver operating characteristic curve.

^c^DHTT: digital health technology tool.

^d^AI: artificial intelligence.

^e^QOL: quality of life.

^f^RCT: randomized controlled trial.

^g^PSS-10: Perceived Stress Scale.

^h^GAD-7: Generalized Anxiety Disorder–7

^i^PHQ-9: Patient Health Questionnaire.

^j^AUC: area under the curve.

^k^UCSD: University of California, San Diego.

## Background: A Shared Lexicon

To begin discussions, participants shared their perspectives on some of the terminology relevant to this emerging area of research. In Table 2, we restate some of the key points raised to orientate readers in the following report.

**Table 2:** Key terms relevant to the discussion. Participants shared terminology relevant to this emerging area of research.

Term	Definition	References
Multimodal measures	Referencing “Multimodal Deep Learning,” multimodal measures are derived from multiple input modalities (eg, activity, sleep, heart rate, patient-reported outcomes, or contextual data)	[24]
Health-related quality of life	An individual’s or a group’s self-perceived physical and mental health over time	[25]
Digital measure	Sensor-derived objective measures arising from “connected digital products.” Includes active tests captured via a mobile platform and continuous passive data collected from a wearable technology but excludes electronic patient-reported outcomes and other subjective measures collected from mobile platforms. An all-inclusive term, encompassing all stages of maturity, settings, and technologies.	[26,27]
Digital end point	A subset of robustly evaluated digital measures that have successfully pursued acceptance or qualification and can be used as decision-making evidence in clinical trials	[27]
Digital biomarker	Objective quantifiable physiological and behavioral data that are collected and measured by means of digital devices such as portables, wearables, implantables, or digestibles. The data collected are typically used to explain, influence, or predict health-related outcomes.	[27]
Patient-reported outcome	Assessments about how patients feel or function in their daily lives where the information is reported by the patient themselves, without interpretation or modification by someone else. Note that assessments can cover a wide range of relevant categories, some of which are more quantifiable and less subjective (including medication use or symptom presence), and some which are more subjective (including symptom severity and perception of well-being).	[26]

## Session 1: Disease Detection

The first session focused on the use of multimodal data and machine learning for disease detection. Detecting deviations from normal behaviors and processes is a key step in triggering further actions, whether that be a follow-up with a health care provider or a direct digital intervention [28-30].

The session started with a keynote from author LF of Evidation Health, who spoke about how person-generated health data (PGHD; adapted from [31]) are transforming public health applications of disease detection. Author LF provided an overview of how PGHD are being used to detect and measure disease progression in a range of indications, the use of PGHD for detecting COVID-19, and the challenges of distinguishing COVID-19 from other influenza-like illnesses and infections [7]. LF underlined that machine learning model performance needs to be evaluated with a specific context of use in mind and that, without such context, model performance is ultimately of little relevance in terms of clinical utility and large-scale adoption. Finally, the keynote closed with a discussion of how what we classically think of as *evidence* can be a source of value to patients themselves, by helping them manage and understand their own health.

The following talks covered a wide range of indications, including author FL of Roche who discussed the use of smartphone-based apps to monitor neurological conditions including Parkinson disease [9] and multiple sclerosis [10,32]. Author BV shared work from the Byteflies platform showing how the system is being deployed for longitudinal monitoring of sleep disorders and cardiorespiratory and neurodegenerative conditions, and for detecting seizures in epilepsy [11,12]. Finally, author GG presented recent work examining how unsupervised measurements of autonomic nervous system signals, including photoplesmography and electrodermal activity (EDA), are showing value in the detection and staging of mental health conditions like anxiety and depression, and how these measures play a complementary role to traditional biomarkers, becoming a useful tool in enhancing clinical trials and precision psychiatry [13-15].

The session was closed with a short panel discussion featuring all the speakers that focused on questions raised by the attendees. One question addressed the pros and cons of data collection via *bring your own device* (BYOD; ie, allowing participants to connect their own devices) versus data collection via an app versus provisioned devices. The speakers agreed that there are different advantages to each approach. For example, BYOD enables comparison to a personal baseline and has advantages for device adherence, whereas provisioned devices can enable higher data uniformity and eliminate barriers to participation due to lack of access to appropriate hardware. Overall, the key is to select the right data collection approach for a given setting; where data consistency or a specific data type or density is priority, for example, in a smaller randomized controlled trial, provisioning may be preferred [33]; BYOD may in turn be preferred in settings where scale becomes limiting or where long-term “pervasive” monitoring places an emphasis on measuring ecologically valid natural behavior [34]. It was noted that while progress has been made around BYOD for patient-reported outcomes [35-37], similar progress for digital measures has not been seen and will be a key step in unlocking the value previously outlined. Another question focused on challenges to integrating objective (ie, from wearable devices) and subjective (eg, from surveys of patient-reported outcomes) inputs. The panel pointed out that many disease detection applications combine both objective and subjective inputs, for example, asking participants to confirm a signal or get a follow-up test. They also pointed out that subjective and objective inputs measure different aspects, so we should not expect them to correlate; however, this also means that they may have different relationships to a given concept of interest [38]. Thus applications that combine objective and subjective inputs can have an advantage in signal detection for disease detection. To help clarify this point, consider the following example on general well-being: a range of objective characteristics can be measured that are informative of overall well-being, including social media activity, patterns of sleep and activity, news consumption, patterns of independence, and many other objective data sources; these sources are informative of several aspects of subjective well-being (eg, perception of health), but none have a direct relationship to any specific aspect of subjective well-being, and what relationship there is differs between individuals [39].

There were also questions on the value of specific objective features (eg, EDA in stress), and GG discussed how this is a special case because this objective marker directly measures autonomic nervous system activation and thus gives a very good signal on psychological state. This was contrasted against other objective measures (eg, step counts) that have a more indirect relationship to symptoms like depression and anxiety.

The panel also discussed the impact of covariates within a cohort (eg, comorbidities) and how it influences model performance. Specifically, when trying to derive more “generalizable” models, which perform well across a broad range of unseen individuals, there is a need to incorporate a large number of covariates, and these covariates can have highly varying relevance across individuals. Progress on this topic has been made in other fields [40], but it was noted that such considerations are particularly relevant to multimodal measures.

## Session 2: Measuring Well-being

The second session focused on measuring QOL and well-being. This ever-growing field has seen proof of concepts for measures across a range of health-related QOL-relevant symptoms, including fatigue [1,41], depression [3,42,43], stress [2], anxiety [44], and independence [8,45], and significant resource is being invested to understand what “wellness” means for diverse populations [46].

Author AS of Rice University started the session with a keynote on *multimodal sensor data analysis and modeling for health and well-being*, discussing her vision for how measures can underpin decision support and behavior change interventions. Her examples included schizophrenia [47], mood, and stress [16]. She also discussed challenges in in-the-wild multimodal data modeling such as model personalization and adaptability to new users/patients and missing data.

Author BS of the University of California, San Diego then discussed current limitations in generalizability [48]. He proposed that the growth of personal sensor devices should enable us to augment classification by demographics and genetics, by including time series of physiology and behavior in our understanding of human diversity [17,18,49]. BS suggested developing algorithms that account for these dynamical differences, especially in health and wellness settings. Author FC of Cambridge Cognition then presented her work on the measurement of fatigue, which is increasingly understood to be a highly patient-relevant symptom across a large range of conditions [50-53]. She discussed the heterogeneity of the manifestations of fatigue, and their approach to combining active tests, voice biomarkers, and passive data collection to capture this complex symptom. Author CvH of imec then presented his work on digestible sensors and sensorized toilets for examining gut physiology. Finally, author SF of the Massachusetts Institute of Technology presented work on digital signals from wearables and smartphones, with application for the assessment of depression symptoms [22] and suicidal thoughts [23].

Overall, the panel discussion focused on measuring well-being as a whole versus specific aspects of QOL. While developing measures for specific aspects remains highly relevant for clinical development and treatment—for example, a measure of anxiety severity enables drug development and management of diagnosed individuals—personalized measures of general well-being have substantial application in public health and in engagement with individuals’ prediagnosis. Advancements to date have focused more on the former, as the relevance to the pharmaceutical industry is higher and the validation pathway is simpler [27]. The former also limits the diversity of experience captured and so frames an opportunity for reconceptualizing wellness, health, and QOL derived from broader participation in mapping individuals’ perceived needs. The panel also discussed whether it is possible and valuable to stratify mood predictions (ie, creating *semipersonalized* models where individuals with similar manifestations, personas, or journey stages are grouped together). Stratification based on objective behavioral data and digital signals can also advance our understanding of a condition by delineating commonalities across patients.

## Key Discussion Points

### Value of Multimodal Measures

Multimodal digital measures have expanded the number of possibilities for new ways to measure health by capturing an increasing number of proxies for multiple aspects of functions related to health. The panel emphasized that such measures are not a replacement for patient-reported outcomes but additional, complimentary tools to help understand the patients’ lived experience, ideally in a low burden and unobtrusive way. The research priority should therefore focus on measures that matter when defining patients’ health or general wellness. To achieve that, a 4-level sequential framework has been recently proposed by Manta et al [54] to evaluate meaningfulness of digital measures, namely, meaningful aspects of health, defining the aspect of a disease to address; specific and targeted concept of interest; outcome to be measured; and end point, including methodology and analysis plan to estimate patient improvement (eg, due to treatment).

While the majority of the research efforts are focusing on the definition and development of outcome measures, the adoption and investigation of these outcome measures in clinical trials as exploratory assessments is key to the development and validation of end points. The panel highlighted the rapidly expanding range of digital cognitive decline measures as an example and the need for the field to do more comparative studies [3] and patient-centric research [54] to focus efforts around the most meaningful and valuable candidate measures. Personalized or individual health trajectories were highlighted as potentially highly valuable, both to patients and to stakeholders outside of clinical development, for example, payer organizations exploring value-based agreements. Personalized health trajectories will require the possibility to define multiple health measures of interest, as no single measure will be equally relevant across individuals and across individual health journeys [54]. LF pointed to a key enabler being access to “healthy” data via monitoring of individuals prior to key events or diagnoses such that individualized baselines and, subsequently, individualized responses can be observed [8].

In the past decade, and accelerated by the widespread use of smartphones and other connected digital products, the use of digital products and devices in clinical trials has grown substantially, albeit primarily in observational studies and non–industry-funded clinical trials focusing on wellness [55]. The COVID-19 pandemic has by necessity further accelerated the adoption of digital health solutions for clinical research in the context of remote monitoring and telehealth [56].

Examples of the most advanced clinical applications of multimodal digital data are in Parkinson disease [57-60] and multiple sclerosis [60,61] with focus on motor function; cognitive decline in Alzheimer disease [62]; and diagnosis of depression [3], Friedreich’s ataxia [63], chronic obstructive pulmonary disease [60,64], and COVID-19 [7,65,66]. Interest in multimodal digital measures is also growing among early drug discovery researchers, where personalized medicine approaches can be enabled by capturing longitudinal information on patients behaviors and in real-world settings, sometimes referred to as “digital phenotyping” [67-70]. Indeed digital measures are seen as a new component of real-world data [71]; thus to drug discovery stakeholders, multimodal measures can also serve an important role by helping to bridge the gap between evidence generation in clinical development and late-phase studies.

### Challenges Remaining

As the number of technologies and sources of digital health data increases, data integration and harmonization remain open challenges. The panelists identified three key obstacles that will need to be overcome to maintain momentum in the field.

First, slow and limited collaborative efforts in prioritizing data sharing will continue to hold back at-scale development and evaluation of novel digital measures and end points. Many companies are starting to realize the value of data sharing internally to their own walls [67], and increasingly, Findable, Accessible, Interoperable, Reusable data principles are becoming a core part of many data strategies [72]. Collaborations like the Innovative Medicines Initiative project RADAR-BASE [73-75] and the subsequent impact on a range of projects and application areas point to a possible path forward and the impact that precompetitive work in this space can have on productivity. Furthermore, multimodal sensor data is currently lacking broadly accepted and adopted common data models [76], which follow the example of other data types such as genomics and electronic health records, and have been a catalyst for progress in those fields; progress is being made [77], but more needs to be done to drive broad adoption [78]. Progress here will facilitate data integration, synchronization, and fusion that are often significant technical challenges at the individual study level when aligning and analyzing a network of connected devices [79]. A consequence of this is that substantial resources must be dedicated to technical challenges, slowing overall progress and innovation. The impact of better alignment on standards can be seen, for example, in the impact the Clinical Data Interchange Standards Consortium (CDISC) [80] has on submission data; thus it is important that collaborative efforts to make CDISC-compliant adaptations for digital health data are making progress [81]. Equally, progress on Fast Healthcare Interoperability Resources specifically on global standardization of data formats for digital health applications is encouraging [82].

Lastly, the rapid evolution of the digital health market and the short life cycle of wearables and connected devices [83,84] are challenges for data integration and reproducibility and generalization of analytical methodologies at the basis of digital measures and end points. Scaling innovation and efficient evaluation of new technologies and updated versions of hardware and software will require adherence to modular evaluation frameworks [85].

Future advances are expected from cross-industry initiatives to develop data platforms such as the Digital Medicine Society sensor integration initiative [78].

### Conclusions and Path Forward

With the future of health care in mind, the panelists touched on a broad range of key takeaways. It is critical to incorporate practical, representative, and systematic approaches to involving patients in everyday health decisions [14]. Several examples discussed highlighted the importance of decision support systems or outcomes for clinical development and the value of early engagement with regulators in this space [86]. The panelists also discussed the significance in bridging the gap from measures to medicine: clinician confidence. Multimodal measures and continuous data capture are new concepts and have not been used by many practitioners, but these methods have the ability to contextualize observations and provide a direct connection to patients.

The workshop focused on sharing experiences and perspectives in the expanding use of multimodal data (multiple simultaneously collected objective data modalities, contextual information, and subjective inputs) to detect disease and capture complex outcomes. Across a wide range of examples, from infectious diseases to mental health and well-being, the speakers showcased the progress made and expressed optimism for future advancement and progression in the field.

## Abbreviations

BYOD: bring your own device
CDISC: Clinical Data Interchange Standards Consortium
EDA: electrodermal activity
PGHD: person-generated health data
QOL: quality of life
